# Aspirin, but Not Tirofiban Displays Protective Effects in Endotoxin Induced Lung Injury

**DOI:** 10.1371/journal.pone.0161218

**Published:** 2016-09-01

**Authors:** Jessica Tilgner, Klaus Thilo von Trotha, Alexander Gombert, Michael J. Jacobs, Maik Drechsler, Yvonne Döring, Oliver Soehnlein, Jochen Grommes

**Affiliations:** 1 European Vascular Center Aachen-Maastricht, Rhenish Westphalian Technical University Aachen, Aachen Germany; 2 Maastricht University Medical Center, Maastricht, The Netherlands; 3 Institute for Cardiovascular Prevention, Ludwig Maximillian University Munich, Munich, Germany; 4 Department of Pathology, University of Amsterdam, Amsterdam, The Netherlands; 5 German Centre for Cardiovascular Research (DZHK), Partner Site Munich Heart Alliance, Munich, Germany; Hospital for Sick Children, CANADA

## Abstract

**Background:**

Treatment of acute lung injury (ALI) remains an unsolved problem in intensive care medicine. Recruitment of neutrophils into the lungs, regarded as a key mechanism in progression of ALI, depends on signaling between neutrophils and platelets. Consequently we explored the effect of platelet-targeted aspirin and tirofiban treatment in endotoxin induced acute lung injury

**Methods:**

C57Bl/6 mice were exposed to aerosolized LPS (500μg/ml) for 30min and treated with Aspirin (100μg/g bodyweight via intraperitoneal injection, 30 min before or 1 hour after LPS inhalation) or Tirofiban (0.5μg/ g bodyweight via tail vein injection 30 min before or 1 hour after LPS inhalation). The count of alveolar, interstitial, and intravascular neutrophils was assessed 4h later by flow cytometry. Lung permeability changes were assessed by FITC-dextran clearance and protein content in the BAL fluid.

**Results:**

Aspirin both before and after LPS inhalation reduced neutrophil influx into the lung and lung permeability indicating the protective role of Aspirin in ALI. Tirofiban, however, did not alter neutrophil recruitment after LPS inhalation. Release of platelet-derived chemokines CCL5 and PF4 and neutrophil extracellular traps was reduced by Aspirin but not by Tirofiban.

**Conclusion:**

Aspirin, but not Tirofiban reduces neutrophil recruitment and displays protective effects during endotoxin induced lung injury.

## Introduction

Although considerable progress has been made in understanding pathophysiology of acute lung injury (ALI) and despite all innovations in intensive care medicine, the mortality of ALI remains up to 40% with an age-adjusted incidence of 86.2 per 100.000 person-years [1]. Since the original description of the acute respiratory distress syndrome (ARDS) in 1967, which is the severest form of ALI, mortality and morbidity of ALI and ARDS have been reduced by lung-protective ventilation and fluid-restrictive management [2]. ALI and ARDS are characterized by an increased permeability of the alveolar-capillary barrier resulting in lung edema with protein-rich fluid, thus resulting in impairment of arterial oxygenation. ALI/ARDS is defined as a lung disease with acute onset, non-cardiac, diffuse bilateral pulmonary infiltrates and a paO_2_/FiO_2_ ≤ 300 for ALI or a paO_2_/FiO_2_ ≤ 200 for ARDS. A recent report recommends use of three categories of ARDS, based on the degree of hypoxemia: mild (200 mmHg < paO_2_/FiO_2_ ≤ 300 mmHg), moderate (100 mmHg < paO_2_/FiO_2_ ≤ 200 mmHg), and severe (paO_2_/FiO_2_ ≤ 100 mmHg) [3].

Bacterial or viral pneumonia are major causes for development of acute lung injury. Sepsis due to non pulmonary infections, aspiration of gastric contents, major surgery or trauma can also induce the injury [2,4]. LPS inhalation mimics human Gram-negative ALI, inducing neutrophil recruitment, pulmonary edema and finally impairment of gas exchange [5]. Recruitment of neutrophils is a key event in development of ALI [4] leading to plasma leakage and deterioration of oxygenation. The importance of neutrophils in ALI is supported by studies where lung injury is abolished by depletion of neutrophils [6,7]. Much of the neutrophil-dependent ALI is mediated by granule proteins released from activated neutrophils. For example, azurocidin and α-defensins were implied to directly alter permeability changes [8,9] whereas proteases of neutrophilic origin such as neutrophil elastase have been suggested to be important in degradation of surfactant proteins, epithelial cell apoptosis, and coagulation [10,11]. As a consequence, modulation of this dysregulated immune response seems to be a promising therapeutic target. Moreover, drugs with pleiotropic anti-inflammatory effects, such as simvastatin or pioglitazone display protective effects in ALI due to reduced recruitment of neutrophils [12,13]

Platelets exhibit a central role in primary hemostasis, but they also display important pro-inflammatory roles. After activation, platelets can release and present various molecules indicating their important role in the recruitment of inflammatory cells. The interaction of platelets with neutrophils is mediated through P-Selectin, β_2_ and β_3_ integrins all of which promotes the recruitment of neutrophils. Signaling between neutrophils and platelets is important in models including transfusion-related lung injury, ventilator-induced lung injury, acid-induced ALI or sepsis [14–16]. In this context, previous studies have revealed an important role of neutrophil extracellular traps (NETs) in lung injury [16–19]. NETs are pro-inflammatory, antimicrobial structures consisting of extracellular chromatin decorated with granular and cytoplasmic proteins and are released by activated neutrophils. Interaction of platelet-derived chemokines can induce NETosis [16] and might function as a therapeutic target in acute lung injury[20]. Taken together, in several ways platelets have evolved as amplifiers of leukocyte recruitment in acute lung injury. Therefore strategies to reduce platelet activation in endotoxin induced lung injury might be a promising therapeutic target. Hence, we here tested the effect of platelet-targeted aspirin and tirofiban on lung neutrophil recruitment, lung edema formation, and lung damage.

## Material and Methods

### Animals

Male C57Bl/6 mice, 8 weeks of age, were obtained from Janvier SAS (Le Genest Saint Isle, France). Mice were housed in our institute for laboratory animals. All the animals were kept under standardized conditions: temperature between 22°C and 24°C; relative humidity 50–60%; 12 h of light following 12 h of darkness. The animals had free access to food and water. The local ethical authorities approved all experiments (Landesamt für Natur, Umwelt und Verbraucherschutz Nordrhein Westfalen AZ 84–02.04.2013–3062). C57Bl/6 mice were exposed to aerosolized LPS (500μg/ml) for 30min and treated with Aspirin (100μg/g bodyweight via intraperitoneal injection, 30 min before or 1 hour after LPS inhalation) or Tirofiban (0.5μg/ g bodyweight via tail vein injection 30 min before or 1 hour after LPS inhalation). In contrast to other models aof acute lung injury, we did not observed mice becoming severly ill after LPS inhalation and no animals died before the experimental endpoint.

### Murine model of LPS induced acute lung injury

Aerosolized LPS from *Salmonella enteritidis* (Sigma Co., St. Louis, MO) dissolved in 0.9% saline (500 μg/ml) was utilized to induce neutrophil-infiltration into the lung. Six mice were exposed simultaneously to aerosolized LPS in a custom-built box (22 cm in length; 10 cm in diameter) connected to an air nebulizer (MicroAir, Omron Healthcare, Vernon Hills, IL) for 30 minutes. Control mice were exposed to saline aerosol (n = 8). Neutrophil counts in bronchoalveolar lavage (BAL) and lung tissue (interstitium and pulmonary vasculature) were assessed 4 hours after inhalation. 30 min before euthanasia, 5 μl FITC-Ly-6G (Gr1) (eBioscience) and 100μl Fluorescein isothiocyanate–Dextran (30 mg/ml FITC-Dextran; 70 kDa, Sigma-Aldrich) were applied by tail vein injection to label intravascular neutrophils. The mice were anesthetized with an intraperitoneal injection of ketamine (125 mg/kg body weight; Sanofi-Cefa GmbH Düsseldorf, Germany) and xylazine (12.5 mg/kg b.w.; Phoenix Scientific). The trachea was dissected and cannulated (PortexFineBore Polythene Tubing, 0.28 mm inner diameter (ID)/ 0.61 mm outer diameter (OD), Smiths Medical International, Keene, NH). 5 x 0.5 ml PBS was injected and withdrawn. Thereafter, the ribcage was opened by a midline incision and euthanasia was done by exsanguation. The pulmonary vasculature was rinsed with 10 ml ice-cold PBS. The lungs were removed, minced and digested with liberase (1:20; 25 mg Liberase RI/ml aqua, Roche Mannheim Germany). Digested lungs were passed through a cell strainer (30 μm; MiltenyiBiotec GmbH, Bergisch Gladbach, Germany) and the resulting single cell suspension was centrifuged for 5 min at 300 g. The pellets were resuspended in 1 ml hank’s balanced salt solution with 0.3 mmol / l EDTA and 0.1% BSA. The bronchoalveolar lavage (BAL) fluid was centrifuged for 5 min at 300g (S1 Fig).

### Flow cytometry

Cell pellets were labeled with PerCP-Cy5.5 anti-mouse Ly-6G, PE anti-mouse CD115, APC-Cy7 anti-mouse CD45 and APC anti-Mouse F4/80 (all eBioscience). Neutrophils were identified by their typical appearance in the forward scatter-side scatter and as CD45^+^, CD115^–^ and PerCP- Gr1^+^ cells (S1 Fig). Within the lung, FITC-Gr1 antibody was used to distinguish between interstitial neutrophils (CD45^+^, CD115^–^, PerCP- Gr1^+^, FITC-Gr1^-^) and intravascular neutrophils (CD45^+^, CD115^–^, PerCP- Gr1^+^, FITC-Gr1^+^). All flow cytometry studies were performed using a BD FACSCanto II (Becton Dickinson, San Jose, CA) and data were analyzed using FlowJo software (Tree Star, Ashland, OR).

### Lung Permeability

FITC-Dextran (70 kDa, Sigma-Aldrich) was used to assess vascular leakage. 100 μl FITC-Dextran (30mg/ml) were administered by tail vein injection 30 min prior to euthanasia and dye extravasation was used to assess change in vascular permeability. The fluorescence of the 100 μl BAL supernatant (Fluo_BAL_) and of 50 μl serum (Fluo_Serum_) was measured and permeability volume was expressed in microlitre (V_Perm_ = (Fluo_BAL_ / 100 μl) / (Fluo_Serum_ / 50 μl) * BAL volume)

### Protein concentration of the BAL

The protein content of the BAL supernatants was assessed using the Bio-Rad Protein Assay based on the method of Bradford (Bio-Rad Laboratories Germany). Measurement of absorbance at 595 nm was performed with a microplate reader (infinite 200,Tecan Group Switzerland).

### Histology

After completion of the experiment, one part of the right lung was fixed in formalin, embedded in paraffin and stained with Mayer’s haematoxylin and eosin for histological examination. Scoring of histological changes was done in compliance with the recommendation of the American Society [21].

### Enzyme-linked immunosorbent assay (ELISA)

CCL5 and PF4 plasma concentrations were assessed was using specific ELISA kits (CCL5 (RayBiotech Norcross, USA), PF4 (Cloud Clone Corp Houston Texas USA) delivered by antibodies-online Aachen, Germany). Measurement of absorbance at 450nm nm was performed with a microplate reader (Sparkcontrol, Tecan Group Switzerland).

### Neutrophil extracellular traps (NET) formation

NET structures were measured in mouse plasma and supernatant of the lung using MPO-DNA ELISA as described previously [16,17]. A 96 well plate was coated with 50 μl/well MPO ab 5 μg/ml 4°C overnight. After washing 3 times (200μl each), 20μl of samples was added to the wells with 80 μl immunoreagent (Peroxydase labeled DNA antibody and anti-Histone-Biotin ab (cell death ELISA plus, Roche) and incubation buffer) for 2 hours with shaking (300 rpm). After washing (3 times) peroxidase substrate was added. After 20 min, absorbance was measured at 405-nm wavelength with a microplate reader (Sparkcontrol, Tecan Group Switzerland). Values are presented as percentage increase of absorbance in comparison to the control group.

### Statistics

All data are expressed as mean ± SD. Statistical calculations were performed using GraphPad Prism 5 (GraphPad Software Inc.). Ordinary one-way Anova followed by Bonferoni analysis were used. * indicates a *p*-value < 0.05.

## Results

### Aspirin reduces neutrophil recruitment in the lung and protects from neutrophil-dependent ALI

**Fig 1:**
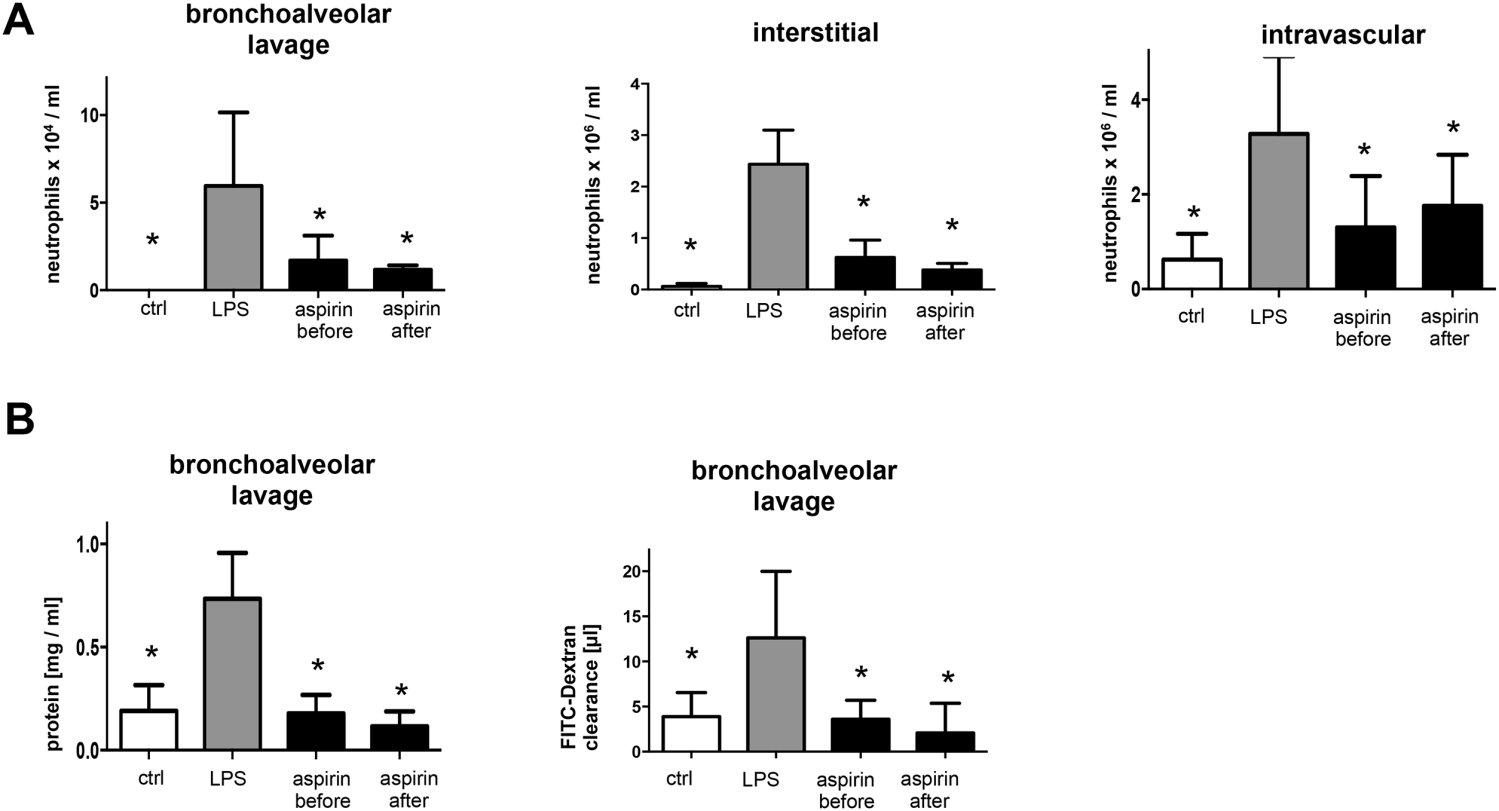
Aspirin reduces LPS-induced acute lung injury by interference with neutrophil recruitment. Mice were challenged with LPS via inhalation and sacrificed 4 hours later. Mice were treated with aspirin (100μg/g bodyweight via intraperitoneal injection) 30 min before or 1 hour after LPS exposure as indicated. **A:** Quantification of alveolar (left), interstitial (middle), and intravascular neutrophils (right) in mice treated as indicated. **B:** protein concentration (left) and FITC-dextran clearance (right), in BAL fluids in mice treated as indicated. n = 6–8 for each bar. * indicates significant difference compared to LPS-treated animals.

After C57Bl/6 mice were exposed to aerosolized lipopolysaccharide (LPS), we observed neutrophil recruitment and plasma leakage in the bronchioalveolar lavage fluid (BALF). Treatment with LPS increased the number of intravascular, interstitial, and alveolar neutrophils as analyzed by flow cytometry of lung homogenates and BALF (Fig 1A). Furthermore, the protein concentration as well as the clearance of fluorescent dextran increased in the BALF by LPS treatment indicating enhanced plasma leakage and edema formation (Fig 1B). To test the potential role of aspirin in this model of neutrophil-mediated ALI, mice were treated with aspirin prior to LPS exposure. In these experiments we found that aspirin reduced the recruitment of neutrophils after LPS inhalation in the intravascular, interstitial, and alveolar compartment of the lung (Fig 1A) and prevented enhanced pulmonary vascular leakage indicated by reduced protein content of the BALF and FITC-Dextran clearance volume (Fig 1B). In addition, treatment with aspirin 1 hour after induction of ALI exhibited similar effects (Fig 1A and 1B).

### Histological analyses of lung injury

**Fig 2:**
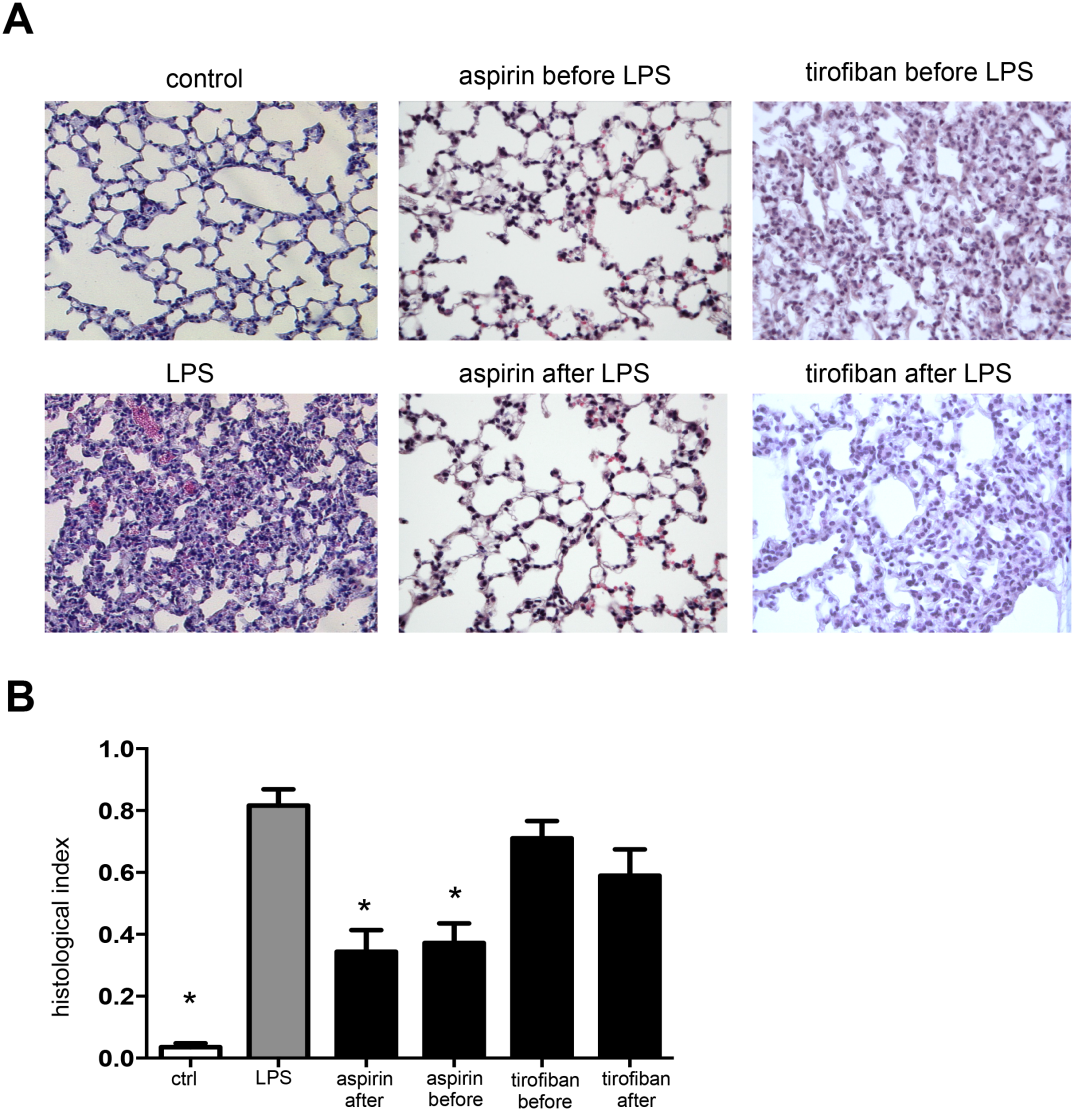
Aspirin, but not tirofiban prevents LPS-induced structural changes in the lung tissue. **A:** Representative histological images of lungs from mice treated as indicated. **B:** Structural analyses of histological lung sections were made on based HE staining. * indicates significant difference compared to LPS-treated animals.

Histological analyses of lung following LPS-exposure revealed alveolar septal thickening, accumulation of inflammatory cells in the interstitial and the alveoli, and influx of protein-rich fluid into the alveolar space as compared to control mice exposed to aerosolized saline solution. Both aspirin administration before and after LPS inhalation abrogated histological alterations of this kind, further supporting its protective role in neutrophil-mediated ALI (Fig 2).

### No Protective effect of Platelet glycoprotein GPIIb/IIIa inhibitor in the endotoxin induced lung Injury

**Fig 3:**
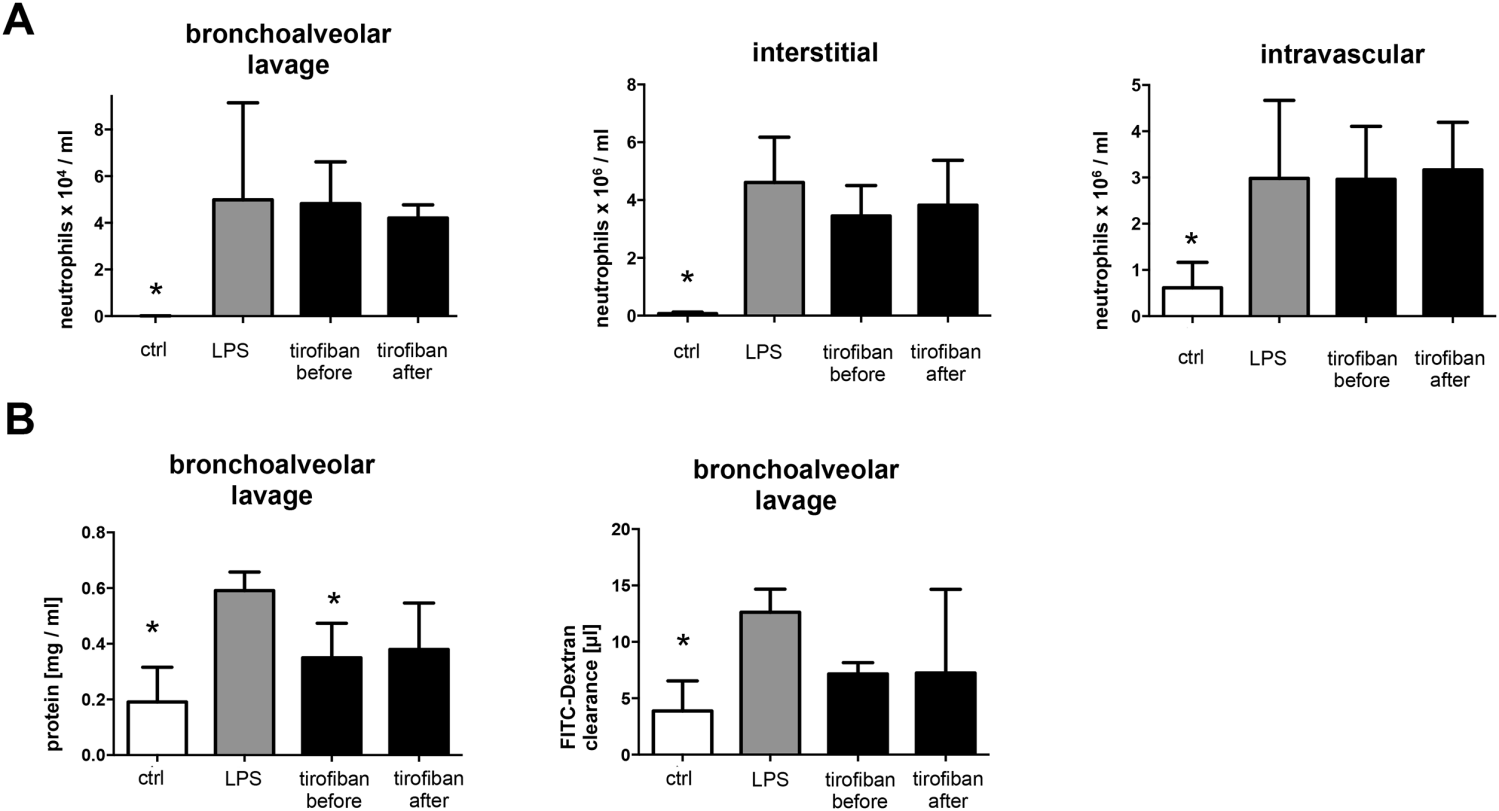
Tirofiban displays no protective effect in LPS-induced acute lung injury. Mice were challenged with LPS via inhalation and sacrificed 4 hours later. Mice were treated with tirofiban ((0.5μg/ g bodyweight via tail vein injection) 30 min before or 1 hour after LPS exposure as indicated. **A:** Quantification of alveolar (left), interstitial (middle), and intravascular neutrophils (right) in mice treated as indicated. **B:** protein concentration (left) and FITC-dextran clearance (right), in BAL fluids in mice treated as indicated. n = 6–8 for each bar. * indicates significant difference compared to LPS-treated animals.

To address mechanisms underlying the platelet-neutrophil axis dependent lung injury by LPS we tested tirofiban -a synthetical non-peptide antagonist of the IIb/IIIa receptor—before and after LPS inhalation. Tirofiban failed to reduce neutrophil recruitment in the interstitial, intravascular and bronchoalveolar space of the lung (Fig 3). Furthermore tirofiban did not exert effects on plasma leakage and edema formation (Fig 3). In addition histological analyses revealed no protective effect of tirofiban treatment (Fig 2).

### Release of platelet-derived chemokines

**Fig 4:**
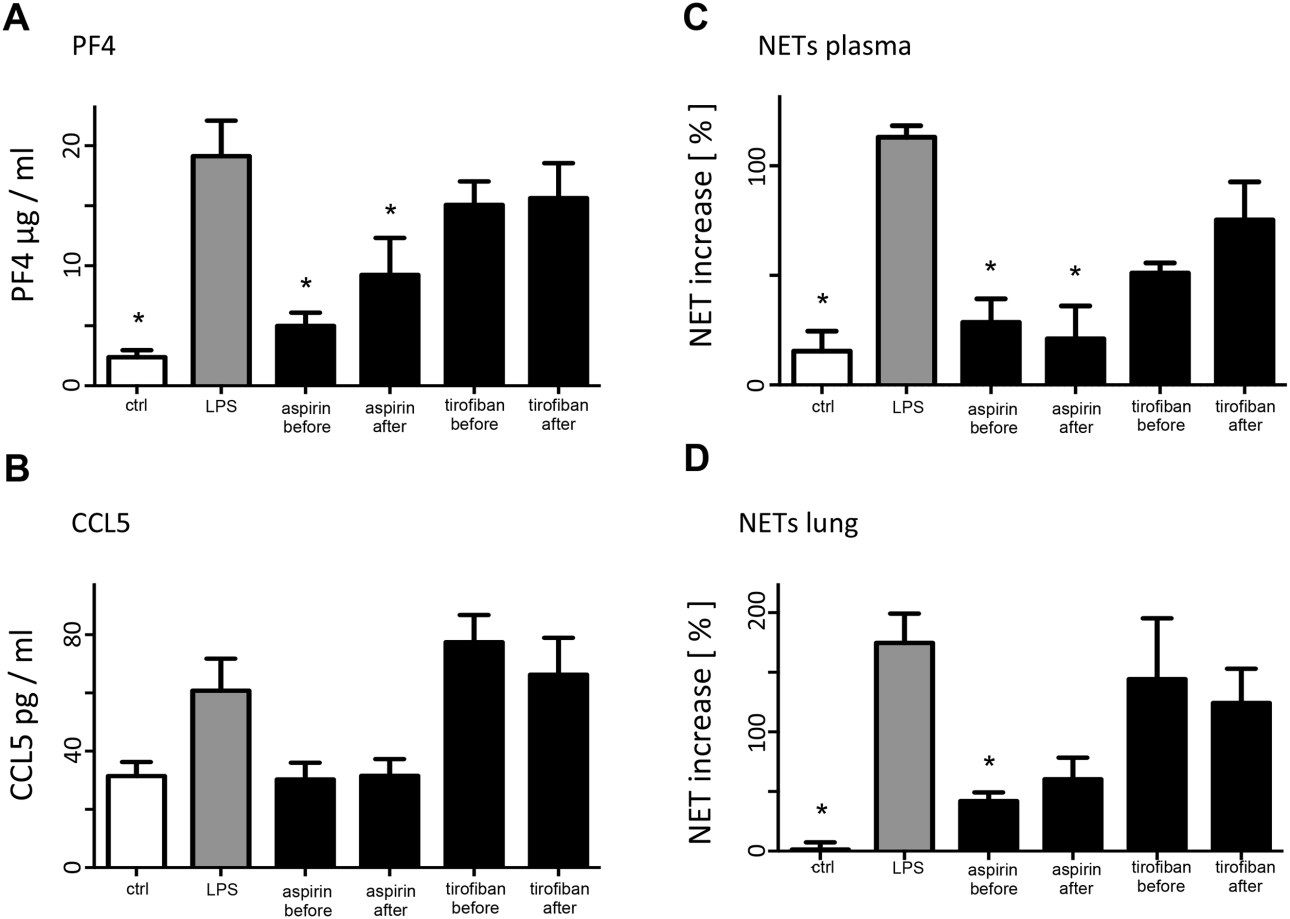
Expression of platelet-derived chemokines (CCL5 (RANTES) and CXCL4 (PF4)) and NET release. Mice were challenged with LPS via inhalation and sacrificed 4 hours later. Mice were treated with tirofiban ((0.5μg/ g bodyweight via tail vein injection) 30 min before or 1 hour after LPS exposure as indicated or with aspirin (100μg/g bodyweight via intraperitoneal injection) 30 min before or 1 hour after LPS exposure as indicated. **A:** Plasma concentration of PF4 (CXCL4) after treatment as indicated. **B:** Plasma concentration of CCL5 after treatment as indicated. **C and D:** NET formation in the plasma (**C**) and supernatant of the lyzed lung (**D**). Values are presented as percentage increase of absorbance in comparison to the control group. n = 6–8 for each bar. * indicates significant difference compared to LPS-treated animals.

We investigated the release of platelet-derived chemokines in endotoxin induced lung injury. As previously shown, interaction of CXCL4 (PF4) and CCL5 (RANTES) increase neutrophil recruitment in ALI [20]. Whereas Tirofiban showed no reduction of PF4 and CCL5 release in comparison to the LPS group, Aspirin reduced the expression of both chemokines. However, only the treatment with Aspirin before LPS inhalation revealed a significant reduction of PF4 release (Fig 4A and 4B).

### NET formation in acute lung injury

LPS inhalation induced NET release in the lung and serum. Treatment with Aspirin, but not Tirofiban resulted in reduced circulating NETs (serum) and in the NETs in the lung tissue (Fig 4C and 4D).

## Discussion

In our study we confirmed a protective role of aspirin, but no role of tirofiban in endotoxin induced lung injury. Together with the protective effect in other models of acute lung injury, our results implicate aspirin as a potential therapy for acute lung injury. Platelet glycoprotein GPIIb/IIIa can interact with neutrophils and hereby stabilizing platelet neutrophil aggregation. However, tirofiban, which is a synthetical non-peptide antagonist to platelet glycoprotein GPIIb/IIIa and has exerted protective effect in TRALI, did not reduce recruitment of neutrophils in our model of acute lung injury.

The recruitment of neutrophils is classically defined as a multistep process including leukocyte rolling, activation, adhesion, and subsequent transmigration, involving cell adhesion molecules and chemokines and their respective receptors [22]. Neutrophil recruitment into the lung is unique and influenced by several factors including neutrophil deformability, adhesion molecules and the unique capillary structure of the lung [23]. Whereas acid induced ALI is largely selectin-dependent [15,24] blocking selectins does not protect against LPS induced ALI [20]. The role of selectins for neutrophil transmigration in the lung depends on the inflammatory stimulus [4]. In contrast, neutrophil β_2_-integrins are of importance in LPS-induced ALI, whereas neutrophil recruitment in acid-induced ALI occurs independently of CD18 [25,26]. Stimuli derived from gram-negative pathogens primarily elicit CD11/CD18-dependent influx of neutrophils, whereas gram-positive products or bacteria attract neutrophils via a CD11/CD18-independent route [25].

Platelets act -beyond their essential role in primary hemostasis- as important effectors in innate immunity and host defense. After activation of platelets at the site of inflammation they can adhere to other platelets, neutrophils or monocytes and hereby recruit neutrophils and monocytes to the site of inflammation [27,28]. In several ways platelets have evolved as amplifiers of leukocyte recruitment in acute lung injury. Zarbock *et al*. revealed that the P-selectin-dependent platelet-neutrophil interaction is essential in acid induced lung injury. Either reducing circulating platelets or blocking P-selection prevents the development of acid induced lung injury. [15]. P-selectin stored in α-granules can be incorporated into the plasma membrane after activation. Hereby, platelets can adhere to PSGL-1 on neutrophils and induce neutrophil activation resulting in activation of neutrophil β_2_-inegrins α_L_β_2_ (LFA-1) and α_M_β_2_ (MAC-1). Both the activated MAC-1, which can bind to GPIb or GPIIb/IIIa on platelets, or LFA-1, which may bind to Intercellular Adhesion Molecule (ICAM)-2 on platelets, can stabilize the cell-cell complex of neutrophils and platelets [18]. These platelet-neutrophil complexes via P-Selectin were regarded as an important interaction between platelets and neutrophils. Although platelet depletion and treatment with aspirin prevented lung injury in a model of transfusion-related acute lung injury (TRALI), blocking the P selectin or CD11b/CD18 pathways did not display a protective effect in this ALI model [14]. In addition, inhibition of the P-selectin displays no protective effects in endotoxin induced lung injury [20].

In LPS-mediated ALI, we unveiled an alternative pathway of platelet-instructed neutrophil recruitment into the lungs. Herein, platelet chemokines CCL5 and CXCL4 (PF4) form heteromers inducing CCR5-mediated neutrophil recruitment [20,29]. In turn, antibodies to the platelet-derived chemokines CCL5 and CXCL4, pharmacological disruption of the CCL5-CXCL4 heteromers or, neutralization of CCR5 diminish lung edema, neutrophil infiltration, and tissue damage in LPS-,acid- and sepsis-induced ALI [16]. Whereas platelet-neutrophil aggregation is important in acid-induced ALI or TRALI [14,15], current studies have revealed the great importance and key role of the platelet-derived chemokines [20]. In this context, Rossaint *et*.*al*. showed that heteromers of CCL5 and CXCL4 induce NET formation during ventilator induced lung injury (VILI) [16]. Previously, Caudrillier *et*.*al*. revealed the important role of platelets to induce NETs in TRALI. In TRALI, treatment with either aspirin or tirofiban—a GPIIb/IIIa inhibitor- decreased acute lung injury and improved survival [17]. In our LPS-induced ALI, aspirin treatment both before and after inhalation displayed similar protective effects. Aspirin treatment reduces platelet activation and hereby displays protective effect in ALI. After platelet activation, thromboxane A_2_ (TXA2) is generated from arachidonic acid via a sequential reaction catalyzed by cyclooxygenase-2 (COX-2). Aspirin inhibits prostanoid synthesis by acetylation of COX-2. Acetylation of COX-2 switches catalytic activity to convert arachonid acid to 15R-hydroxyeicosatetraetonic acid which can subsequently converted to aspirin triggered lipoxin (ATL). Both aspirin as well as ATL protects from lung injury [30]. Therefore we analyzed the release of both chemokines (CCL5 and CXCL4) and NET release in our LPS-induced ALI. Aspirin treatment displayed reduction of chemokine expression and NET release. In contrast to Aspirin, Tirofiban did not influence the chemokines expression or the NET release. Consequently, in our endotoxin induced ALI, we observed no protective role of tirofiban. Interaction of Mac-1 from neutrophils and GPIIb/IIIa presented by platelets can stabilize the cell-cell interaction. In Acid induced ALI, this platelet neutrophil aggregation has been shown to be of great importance [18]. In endotoxin induced ALI, however, platelets use other pathways such as chemokines to induce neutrophil recruitment.

Beside their central role in primary hemostasis, activated platelets can release and present various molecules orchestrating recruitment of inflammatory cells in host defense and additional immunologic functions. However, overwhelming recruitment of neutrophils to site of inflammation carries the risk of harming the host itself. Aspirin pretreatment can reduce platelet activation and hereby abrogating lung injury in different models of ALI. Moreover, treatment early after LPS inhalation was protective implicating a potential role of aspirin in the prevention of ALI.

## Supporting Information

S1 Dataset Acute lung injury. (PZF)

S2 Dataset ELISA (CXCL4,CCL5 and neutrophil extracellular traps). (PZF)

S1 Fig Experimental flow-chart and gaiting strategy during flow-cytometry. (TIF)
