# Supplementary figures and images for: Aspirin, but Not Tirofiban Displays Protective Effects in Endotoxin Induced Lung Injury
Source: PLoS One. 2016 Sep 1;11(9):e0161218. doi: 10.1371/journal.pone.0161218 (PMC5008681; doi:10.1371/journal.pone.0161218)

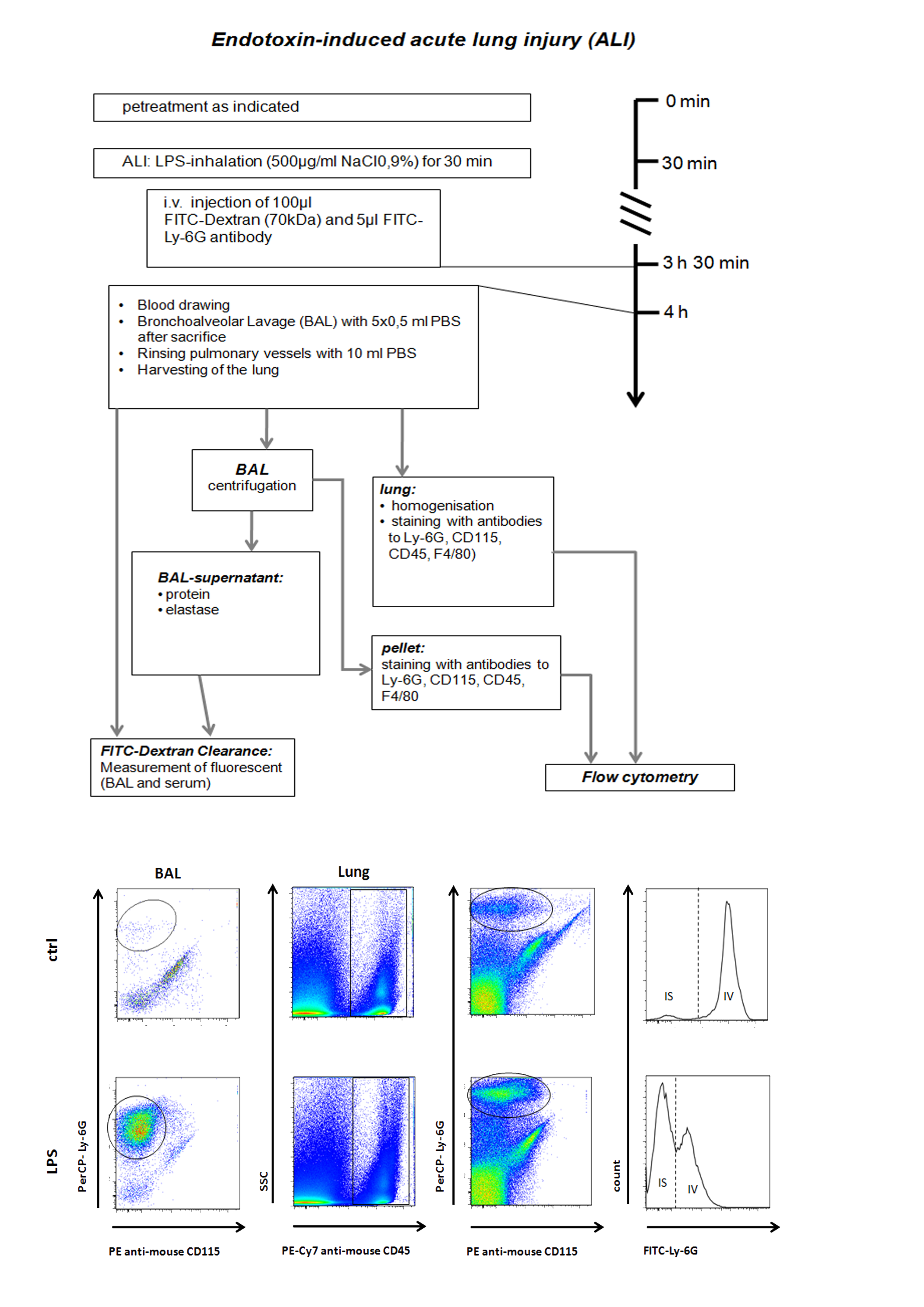

Supplement: S1 Fig — (TIF) [file pone.0161218.s003.tif]
